# Access to trade credit and its impact on the use of European Union (EU) approved pesticides among smallholder cocoa farmers in Ondo State, Nigeria

**DOI:** 10.1016/j.heliyon.2022.e12409

**Published:** 2022-12-21

**Authors:** Ayodeji Damilola Kehinde

**Affiliations:** Department of Agricultural Economics, Obafemi Awolowo University, Ile Ife, Osun State, Nigeria

**Keywords:** Trade credit, EU approved pesticide, Use, Smallholder cocoa farmers, Ondo state

## Abstract

The use of EU-approved pesticides is low in Ondo State, especially among smallholder cocoa farmers. Perhaps, trade credit, being one of the most important sources of credit, would increase the use of EU-approved pesticides among smallholder cocoa farmers. Therefore, this study investigated access to trade credit and its impact on the use of EU-approved pesticides among smallholder cocoa farmers in Ondo State. A multistage sampling procedure was used to select 240 smallholder cocoa farmers for the study. Data were analyzed using descriptive statistics, a double hurdle regression model, and an endogenous switching probit regression model. The descriptive results showed that on average, respondents were 44 years of age, 16 years of farming experience, 7 people for household size, and 2.70 hectares for farm size. Most of the respondents obtain trade credit from their respective farmers' cooperatives (82%), while others obtain trade credit from input suppliers (73%) and cocoa exporters (66%). It was also noted that only 35% (84/240) of the smallholder cocoa farmers use EU-approved pesticides on their cocoa farms. A majority (73.4%) of the respondents used the Ridomin 66WP gold (Cuprous Oxide + metalaxyl-M). Others use Esiom 150SL (Acetamiprid) (37.5%), Funguran-OH (Copper hydroxide) (49.1%), touch down fort (Glyphosate) (47.5%), and Actara 25WG (Thiamethoxan) (25%). The result further showed that age, gender, household size, farm size, cooperative membership, and assets significantly influenced the probability of a farmer receiving trade credit. However, gender, household size, year of farming experience, cooperative membership, and assets are statistically significant in determining the amount of trade credit obtained by the farmers. The first stage of the ESPM revealed that gender, age, household size, farm size, and cooperative membership significantly influence the smallholder cocoa farmers' access to trade credit. While the second stage of the ESPM revealed that significant use of EU-approved pesticides among users of trade credit is affected by age, household size, education, access to extension services, and cooperative membership. Meanwhile, among non-users of trade credit, variables such as age, farming experience, farm size, land ownership, and cooperative membership significantly affect the use of EU-approved pesticides. After controlling for observed and unobserved covariates, the study concluded that access to trade credit positively impacts the use of EU- approved pesticides among smallholder cocoa farmers. It was concluded that policy strategies aimed at increasing the use of EU-approved pesticides among small cocoa farmers should take into account trade credit.

## 1 Introduction

Cocoa (*Theobroma cacao*) is among the most popular cash crops in Nigeria and its production constitutes a significant integral component of the smallholder farming systems of Southwestern, Nigeria (Kehinde, 2021). This region, which includes Ondo, Osun, Ekiti, Ogun, and Oyo, hosts important cocoa farming communities in Nigeria. Ondo State is the leading State in cocoa production in Nigeria with about 77,000 tons in 2019/2020 season only (NBS, 2020). In this regard, cocoa production has gone a long way to reducing the high rate of unemployment in the State. It provides employment and income generation opportunities for over 200,000 people along its supply chain (Kolawole et al., 2020; Adeyemo et al., 2020; Kehinde and Ogundeji, 2022a). In addition, earnings from cocoa production constituted an important source of internal revenue for the government of the producing States to embark on some of their projects and activities (Fadipe et al., 2012). However, in recent years, cocoa production has decreased which has led to a consequential effect on the Nigerian economy (Kehinde, 2021). The decline has particularly affected the socioeconomic development of the cocoa producing States. The decline in cocoa production is attributed to a myriad of problems such as the ageing of cocoa farmers and cocoa trees, depletion of soil fertility, high pest and disease infestation, poor maintenance of cocoa farms, and lack of credit access, among others (Oluyole et al., 2013; Kehinde and Adeyemo, 2017; Kolawole et al., 2020; Kehinde and Tijani, 2021a; Kehinde and Ogundeji, 2022a). One of these issues, and a significant one for Ondo State's cocoa output, is the prevalence of cocoa pests and diseases (Akinneye et al., 2018). The prevalence of pest and diseases attack has led to the loss in cocoa yield, the value of foreign exchange for cocoa beans, farmer's income, and government revenue (Tijani, 2006; Bateman, 2008; Kehinde and Tijani, 2021a). For instance, about 30-45% of cocoa production losses arise from the incidence of diseases and parasites (Tijani, 2006; Kehinde and Tijani, 2021a), which could be up to 80% in some exceptional places with a high infestation (Bateman, 2008).

The efforts to curb cocoa losses arising from pest and diseases has called for the use of pesticides by cocoa farmers. Pesticides are chemical compounds made up of a variety of agrochemicals that are used to fight off diseases and pests that are harmful to the growth and development of crops (Mahmood et al., 2016). The usage of pesticides is becoming more extensive in Nigeria, and it is particularly more concentrated in the cocoa sub-sector. Pesticides are an indispensable method of controlling pests in cocoa production (Akunyili and Ivbijaro, 2006), as between 125,000 and 130,000 tons of pesticides are applied on cocoa farms each year, due to their effective and quick action. The commonly used pesticides on Nigeria cocoa farms are insecticides, herbicides, fungicides, and fumigants. Although pesticides are used solely to improve productivity through reduced or no pest attacks, their use has been connected to unplanned human health consequences (Dankyi et al., 2014; Owombo et al., 2014). There is evidence now that certain pesticides have long-term and extremely harmful consequences on human health (Sarkar et al., 2021). Concurrently, human health problems associated with pesticide use are frequently prominent in developing countries because their farmers frequently lack the ability to read labels with safety warnings (Fianko et al., 2011; Tijani and Sofoluwe, 2012; Sarkar et al., 2021). Many farmers are even unaware of the precise dosage that might reduce the amount of residues that the pesticide leaves on the crop while also being cost-effective. Inappropriate and uncontrollable application of pesticides has the consequence of leaving some residues on the cocoa beans which serve as contaminants in cocoa beans (Asogwa and Dongo, 2009; Fosu-Mensah et al., 2016). These contaminants make cocoa beans dangerous for human consumption, leading to many health problems in human beings. Numerous health issues such as reproductive failures, birth defects, immune system malfunction, Parkinson's disease, and cancers are associated with the consumption of contaminated cocoa beans, while at least 20,000 people die each year (World Bank, 2006; Kehinde and Tijani, 2021a). Pesticide consumption accounts for 20% of the 800,000 suicide deaths worldwide each year (World Health Organization, 2019; Sarkar et al., 2021). In recent times, the high level of pesticide residue, due to its concomitant effect on human health, has adverse effects on the value of cocoa beans coming from Nigeria to the global cocoa market (Adu-Acheampong et al., 2014).

The issue of high levels of pesticide residue has called for a lot of international sanctions in the international market. The EU, Japan, and the USA have suggested the maximum residue levels (MRLs) that cocoa-producing countries must comply with, for their cocoa beans to be allowed into their countries. This is done to lessen the risk of consuming pesticides contaminating food (Bateman, 2010; Faloni et al., 2022). The Food Quality Protection Act of 1996, enacted by the USA Environmental Protection Agency (EPA), indicates the amount of pesticide residues allowed on food products for consumption in the USA (QCCL Annual Report, 2008; Boadu and Boadu, 2021). In addition, the European Union enacted Legislation on MRLs of pesticides (Regulation 149/2008/EEC) of 0.01 mg in September 2008 (QCCL Annual Report, 2008; Boadu and Boadu, 2021). These regulations set maximum levels on the residue of pesticides permitted on imported foods including cocoa beans and consequently ban shipments of cocoa beans that do not meet the minimum residue level (MRL) (International Cocoa Organization, 2008; Boadu and Boadu, 2021). The regulation has reduced the cocoa export from Nigeria which has raised the question of the potential marginalization of African countries in the global cocoa market (Santeramo and Lamonaca, 2019). This is ascribed to the fact that African export performance depends on the average level of Sanitary and Phytosanitary Standards (Bouet et al., 2008). However, the net effect of standards depends on the ability of foreign producers to comply with the more stringent requirements (Beghin et al., 2015; Santeramo and Lamonaca, 2019). As a result, in Nigeria, some pesticides have been banned and some new cocoa-friendly pesticides have been introduced to farmers to minimize residues on cocoa beans. Among the banned pesticides are folar 525, gamalin, Gramoxone, Asulox and Acril DS. However, Actara 25WG, Esiom 150SL, Funfurun-OH, DP champion, Ridomil gold 66WP, Ultimax plus, Kocide to 2000, Touch down round, Round up Clear weed, and Phhostoxin are approved for pest control on Nigeria cocoa farms. Nevertheless, EU-approved pesticides are often too expensive for smallholder farmers and their financing requires liquid cash which is mostly not readily available to smallholder cocoa farmers. In this case, the regulations are considered trade barriers because foreign producers face high costs of compliance (Santeramo and Lamonaca, 2021). Whereas, the vast majority of the smallholder cocoa farmers still have limited access to formal credit because of their inability to cope with the prevailing interest rate and other requirements of formal credit (Adebayo and Adeola, 2008; Ijioma and Osondu, 2015; Oke et al., 2019). The majority of the agricultural development banks that provide credit at subsidized rates have failed to achieve their objective to serve the rural poor. Meanwhile, most rural households continue to rely on the informal credit market to purchase expensive resources such as pesticides, necessary to meet the EU's minimum residue requirement. In this case, trade credit could act as a substitute for conventional credit, particularly for farmers with limited access to bank credit (Mateut et al., 2006; Aderajew et al., 2018). To determine the function of trade credit in agricultural business operations, it is necessary to determine whether it addresses the issue of bank credit rationing, i.e., whether it can take the place of bank loans.

According to many research outputs (McGuinness et al., 2018; Paul et al., 2018; Jory et al., 2020; Tang and Mora, 2020), these two types of finance are either complementing or substitutable. However, one feasible substitute for bank credit is the use of trade credit since bank credit is either too expensive or too challenging to get. Trade credit is an arrangement between a buyer and seller by which the seller allows for delayed payment for its products instead of instant cash payment. One feature that distinguishes trade credit from other sources of credit is that it is a business-to-business agreement in which a customer can purchase goods without paying cash up front, but at a later scheduled date. Due to its specialized nature, trade credit frequently has a short maturity. It relates to either the payment conditions that a supplier gives to its customer or the loan that one company gives to another. With this credit, the debtor can fund its operational expenses while still holding onto liquid assets that will be turned over to the provider on the specified due date. This kind of credit has expenses, or at the very least, opportunity costs, even if it has cash payment conditions. However, for many businesses, trade credit is typically easier to obtain than bank credit. The buyer side of trade credit is the main focus of this study. Thus, in this study, trade credit is an informal (Degryse et al., 2016) and uncollateralized (Troya-Martinez, 2017) lending kind of credit that is enabled by the vendors to lessen the purchasers' financing challenges and enables the acquisition of productive resource (Paul et al., 2018).

Trade credit is one of the most important informal sources of credit and plays an important role in firm financing policy in developing countries, Nigeria inclusive. It arises from delayed payments between individuals that are, the buyer and the seller. The seller gives the buyer credit during this time, and the buyer is responsible for paying back the seller when the payment is due (Le and Cao, 2013). Trade credit is an asset in the seller's accounts receivable while it is a source of funding for the buyer's accounts payable (Ninh and Kieu, 2019). Trade creditors don't need collateral and instead rely on personal connections and relationships, making it possible for those who have been turned down for a loan by banks to get goods for use in their businesses. Trade credit enables customers with limited access to bank credit to obtain the necessary items in an adequate quantity and quality. Due to this fact, trade credit forms the largest source of short-term funds and the most flexible means of financing for business firms. This is further attributed to the fact that most buyers try to delay their payments to sellers to alleviate constraints on credit in a short period. Besides, trade credit lessens the misuse of loans and improves the capacity of the buyer to repay (Burkart and Ellingsen, 2004; Ninh and Kieu, 2019). As a result, the sellers and the purchasers become more dependent on one another. The seller assumes less risk than the lending institutions do. To put it another way, consumers that have restricted access to bank credit use trade credit (Ojenike et al., 2013). In this regard, farmers could make use of trade credit to purchase improved inputs such as fertilizers, pesticides, and seeds among others to enhance their productivity. Given this information, trade credit can be viewed as a significant short-term external financing source that could ensure the use of EU-approved pesticides among cocoa farmers, particularly for smallholders (Le and Cao, 2013), especially during difficult financial times (Tsuruta, 2015a, Tsuruta, 2015b), in order to produce high-quality cocoa beans for sale in the global market.

Despite growing evidence supporting the usefulness of trade credit as a source of business finance, the subject matter is conspicuously understudied in the agricultural sector. There are well-documented studies on the use of trade credit in manufacturing companies (Salima and Kolawole, 2008), the use of trade credit in firms (Ojenike and Olowoniyi, 2012), trade credit and the performance of firms in Nigeria (Ojenike et al., 2013), determinants of trade credit in Nigeria (Ojenike and Olowoniyi, 2014), and trade credit use by shrimp farmers (Ninh and Kieu, 2019). This study is distinct from others in that it examined the factors that determine trade credit and how they affect smallholder cocoa farmers' use of EU-approved pesticides. There is a dearth of information on the subject matter in Ondo State, a place where obtaining bank credit is still difficult since many smallholder farmers lack the collateral necessary to do so, but trade credit makes up a sizeable portion of external funding. In Ondo State, access to external funding in the form of bank loans is a regular problem for farmers. Trade credit is viewed as a crucial short-term financing tool for smallholders to help them overcome issues with bank credit limitations (Cassia and Vismara, 2009; Gama et al., 2010; Garcia-Teruel and Martinez-Solano, 2010). Therefore, this study is conducted to fill the gap in the literature. In line with the pecking-order theory, investigating the impact of access to trade credit on the use of EU-approved pesticides among smallholder cocoa farmers is the main objective to be explored in the study. Specifically, the study describes the socio-economic characteristics of smallholder cocoa farmers; describes the sources of trade credit available to smallholder cocoa farmers; profiles the status of pesticides used by smallholder cocoa farmers; assesses the determinants of trade credit among smallholder cocoa farmers; and determines the effect of access to trade credit on the use of EU approved pesticides among smallholder cocoa farmers. Through the results stemming from this study, the study makes three major contributions as follows: (a) examining the extent of use of trade credit and approved pesticides by smallholder cocoa farmers, (b) establishing the relationship between access to trade credit and use of approved pesticide; and (c) examining the determinants of access to trade credit among smallholder cocoa farmers. This understanding is lacking in the literature, even though it will be useful for policy formulation. This contribution is relevant to creditors, and other society actors, especially farm managers and owners, in addition to academics. The rest of the paper is organized as follows. Section two describes the Literature review. Section three presents the data and the methods used in assessing the determinants of trade credit and its effect on the use of EU-approved pesticides among smallholder cocoa farmers. Section four presents and discusses the empirical findings, while section five concludes.

## 2 Literature review

Cocoa is perhaps the cash crop that contributes the most to Nigeria's economy, especially that of the southwest. Rapid socioeconomic development is brought about by cocoa production and export in the cocoa-producing regions of Nigeria (Kehinde and Adeyemo, 2017). The government used the proceeds from cocoa export as a significant source of finance for important programs including education, healthcare, and the provision of pipe-borne water. However, regrettably, due to significant challenges, Nigeria's cocoa production and income from exporting the product have decreased (Aikpokpodion et al., 2012; Kehinde and Ogundeji, 2022b). Among these difficulties, the proliferation of pests and diseases is one of the primary causes of declining cocoa yields. However, using synthetic pesticides has proven a strategy to keep pests and diseases under control. Unfortunately, the hazards involved with using pesticides have outweighed their potential advantages in reducing hazardous pests (Mahmood et al., 2016). Due to the aforementioned factors, in September 2008, the European Union (EU), which accounts for 85% of cocoa imports from Nigeria and is a significant player in international agricultural trade, created Regulation 149/2008/EEC on Maximum Residue Levels (MRLs) on Pesticides, which established maximum levels on the amount of residue acceptable on cocoa beans (default MRLs 0.01 mg/kg). Pesticide residues are substances that are present in or on products, including, in particular, those that may result from use in plant protection, veterinary medicine, and as a biocide. These residues may include active substances, their metabolites, and/or breakdown or reaction products of active substances currently or previously used in plant protection products.

The term “MRL” refers to the maximum allowable level of pesticide residues in or on food or feed that has been determined in compliance with this rule based on good agricultural practice and the minimal amount of consumer exposure required to safeguard vulnerable consumers (Pigłowski, 2022; Kuchheuser and Birringer, 2022). The EU's harmonized MRLs cover more than 1,300 pesticides that are used in 378 different food products and food groups. A default MRL of 0.01 mg/kg applies to about 690 of these chemicals that are not specifically mentioned in the MRL regulation (Carrasco and Medina, 2021). The EU-coordinated program (EUCP) was formed in 2018 by Commission Implementing Rule (EU) No 2017/660, also referred to as the “2018 monitoring regulation” (Medina and Triacchini, 2020). It is anticipated that cocoa beans with pesticide residue levels above the specified MRL won't be allowed to trade internationally, which could affect the worldwide markets for the agricultural product (Beckman et al., 2020). In samples produced in the EU among the products of plant origin analyzed as part of the 2018 EU-coordinated program, the following non-EU-approved pesticides were reported to have exceeded the legal limit: omethoate, bitertanol, carbendazim (RD), flusilazole, dieldrin (RD), chlorfenapyr, and triadimefon; carbendazim (RD), omethoate, and acephate; (RD). Member States should investigate the erroneous use of pesticides found in particular crops (Medina and Triacchini, 2020). The regulation that was repealed in late 2019 by regulation (EU) 2019/1793 establishes guidelines for the higher level of official controls to be applied to a list of foods and feeds that are not produced using animals but that, because of known or emerging risks, need to be introduced into the EU at a higher level of controls (Carrasco and Medina, 2021). Sixteen insecticides have been given the go-ahead to be used in the production of cocoa in order to comply with EU regulations. However, the majority of cocoa farmers lack the resources to purchase these pricey certified pesticides. Due to two aggravating factors—high-interest rates used by financial institutions and the state of the economy—financing has been highlighted as one of the major barriers to the use of pesticides certified by the EU. The provision of approved pesticides to cocoa producers on trade credit is one approach to tackle this problem.

The body of literature on trade credit and its determinants is expanding. Delays in payments between businesses are the cause of trade credit. Trade credit is a type of short-term borrowing that consequently results from regular business dealings between firms. To examine the significance and effects of trade credit, several academics have looked into its determinants in their respective nations. (Bougheas et al., 2009; Huang et al., 2011; Kestens et al., 2012; Tsuruta, 2015a, Tsuruta, 2015b; Martínez-Sola et al., 2014; McGuinness and Hogan, 2016; Jory et al., 2020; Tang and Mora, 2020). Producers use trade credit due to many reasons. The finance theory contends that trade credit serves as the ideal substitute for formal credit for individuals who require money but are unable to obtain it (Love, 2011; Kabir and Zubair, 2015; Andrieu et al., 2018). Also, trade credit allows the buyer the chance to check the products' quality, which is essential when there is a lot of information asymmetry regarding the quality of the commodities and dishonest behavior is common (Chung and Liao, 2006; Cuñat, 2007). This is the marketing theory. Trade credit provides credit institutions with trustworthy information on the buyer's creditworthiness, which facilitates the buyer's access to formal credit (Wilner, 2000; Wilson and Summers, 2002; Cuñat, 2007). The buyer can also reduce transaction costs and risk by using trade credit (McGuinness and Hogan, 2016; Andrieu et al., 2018; Tang and Mora, 2020). Access to trade credit in agriculture enables farmers to better allocate resources and use them more effectively to increase income (Ninh and Kieu, 2019). As a result, credit-constrained cocoa farmers who are turned down from accessing bank credit due to the risks connected with agriculture have a huge need for trade financing. However, trade creditors must thoroughly vet purchasers based on a number of criteria before extending trade credit to them. The trade creditor must first assess the ability of the farmer to boost his farm's profit. Profit is viewed as a source of investment capital, which increases their capacity to repay trade credit loans.

Also, in order to apply inputs and use effective cultivation techniques to boost yields and pay off debt, the farmer actually needs land capability (Ninh and Kieu, 2019). The trade creditor may sell the land as an asset if the buyer defaults on the loan. Because it is more affordable for them to liquidate collateralized land, the supplier extends more trade credit to farmers who own larger parcels of land. Additionally, a farmer's revenue is influenced by the size of their land, which encourages them to make long-term investments in better production technologies employing both human and financial resources to increase land productivity (Koirala et al., 2016). Also, relationships give the supplier the option to check the honesty of borrowers in order to reduce losses brought on by their dishonesty. Relationships encourage the exchange of commodities and information at various dimensions and intensities of connectivity and openness, which makes trust between people possible (Cuñat, 2007; Shoji et al., 2012; Tsuruta, 2015a, Tsuruta, 2015b; McGuinness and Hogan, 2016; Mogues, 2019). Furthermore, agricultural production involves a number of complex risks for farmers, including those related to financing, marketing, and production. Farming is a lifelong learning process where producers share knowledge with other farmers and learn from their own failures. Such lifelong learning is essential for dealing with uncertainties, such as the return of trade credit, as it contains a thorough understanding of the farm firm. The ability to establish connections with vendors who provide trade credit is also, in theory, correlated with farm firm size. Consequently, it is anticipated that the farm firm's size has an impact on the demand for trade credit (Petersen and Rajan, 1997; Wilson and Summers, 2002; Danielson and Scott, 2004a, Danielson and Scott, 2004b; Huyghebaert, 2006; Giannetti et al., 2011). Because bank loans are less common, smaller businesses are more prone than larger ones to rely on trade credit (Atanasova and Wilson, 2003; Rodríguez-Rodríguez, 2006). However, empirical research by Danielson and Scott, 2004a, Danielson and Scott, 2004b and Niskanen and Niskanen (2006) upholds the finding made by Garcia-Teruel and Martinez-Solano (2010) that larger businesses receive more trade credit financing from their suppliers.

Additionally, suppliers still grant trade credit despite the risk because of the competition. In a market with numerous competitors and providers, a buyer can easily and affordably switch suppliers (Fisman and Raturi, 2004; Fabbri and Klapper, 2016). The seller can feel under pressure to provide customers additional credit out of fear of losing their company. This is especially noticeable in markets for uniform goods, such as those for the pesticides used in the production of cocoa. Contrarily, due to information asymmetry, competition may deprive suppliers of the incentive to establish costly but fleeting connections with buyers, hence reducing the amount of trade credit granted. Businesses that have longer bank loan maturities and stable interest rates significantly demand less trade credit, according to Altunok et al. (2015). Additionally, the fact that a metropolis provides enterprises with more trade credit shows that in markets with increased competition, trade credit can take the place of bank finance (McGuinness et al., 2018; Andrieu et al., 2018; McGuinness and Hogan, 2016; Tsuruta, 2015; Kabir and Zubair, 2015). According to the Pecking Order Theory, bank credit should take precedence over trade credit if it is cheaper for a creditworthy company (Tsuruta, 2015a, Tsuruta, 2015b; McGuinness and Hogan, 2016; Jory et al., 2020; Tang and Mora, 2020). This idea holds that companies with established banking links ask for less trade credit (Ge and Qiu, 2007; Deloof and Van Overfelt, 2011). The reasons for requiring pricey trade credit, such as liquidity management and the minimization of informational asymmetries, cease to be significant once a bank loan is made available. This results in a decrease in the actual demand for trade credit (Molina and Preve, 2012; Wilner, 2000). Credit-rationed businesses are more likely to use and seek trade credit, according to findings of Casey and O'Toole (2014). Hill et al. (2017)'s study revealed that businesses with less access to financial credit prefer to use trade credit financing. Breza and Liberman (2017) also demonstrated that the restriction decreases the likelihood of trade by 11%. The retailer likewise reduces overall purchasing and internalizes procurement to its subsidiaries in response. The inability to grant lengthy trade credit periods might be mitigated, Hill et al. (2017) discovered, via relational contracts. These outcomes appear to be more positive for businesses in emerging markets. Firms with higher profitability and liquidity ratios make fewer requests for trade credit, according to Jaleel et al. (2015). In a different study, Fabbri and Klapper (2016) showed that inventory turnover has a favorable impact on trade credit demand. According to the finding of Hill et al. (2017), companies with insufficient liquidity would request less trade credit.

The effects of trade credit have been examined in numerous studies. A few researchers found that there was a negative relationship between trade credit and corporate financial performance. There are numerous instances of these works, including Martínez-Sola et al. (2014), Sardo and Serrasqueiro (2017), Vo and Ellis (2017), Manrique and Mart-Ballester (2017), Platonova et al. (2018), Xie et al. (2019), Uyar et al. (2020), and Li et al. (2020). However, some studies, including those by Sardo et al. (2018) and Soewarno and Tjahjadi (2020), suggested that trade credit has a positive impact on a company's financial success. Target trade credit is a concern for firms, according to a study by Abuhommous (2017). Additionally, depending on a firm's characteristics, trade credit's effect on profitability varies. The financial justification for trade credit states that larger and more creditworthy businesses will grant trade credit to their smaller clients, increasing the firm's sales and yielding an implicit rate of return. In this regard, bigger and more liquid companies get better returns on trade credit than smaller and less liquid companies. According to the operational incentive for trade credit, businesses with fluctuating demand are expected to lend more trade credit than those with comparatively consistent demand. According to Bougheas et al. (2009), a firm operating with greater trade credits will have a higher cost of production for a given amount of liquidity. Contrarily, Boissay and Gropp (2007) claimed that in order to get out of a difficult scenario with liquidity, businesses should strive to trade credit one-fourth of the goods to their suppliers. In addition, Cunat (2007) asserted that when alternative sources of funding are insufficiently available, rapidly expanding businesses may finance themselves through trade credit. Furthermore, Petersen and Rajan (1997) found that companies with high-profit margins, or those that would profit the most from generating more sales through price discrimination, do in fact have larger accounts receivable. According to Ferrando and Mulier (2012), businesses should extend more trade credit to clients who are momentarily struggling. Since the troubled consumer would otherwise be unable to purchase the items, this also increases their sales. However, businesses will only extend further trade credit if they think a long-term relationship with that consumer will benefit them in the future (Cunat, 2007). As a result, while some studies (Li et al., 2016; Hoang et al., 2019) showed a positive impact of trade credit on a company's value, other studies found no proof of a connection between trade credit and firm performance (Jory et al., 2020). But numerous researchers found the exact reverse. For instance, using data from emerging markets, Orazalin (2019) found a negative association between the volume of trade credits and the firm's profitability.

## 3 Material and methods

### 3.1 Studied area

**Figure 1:**
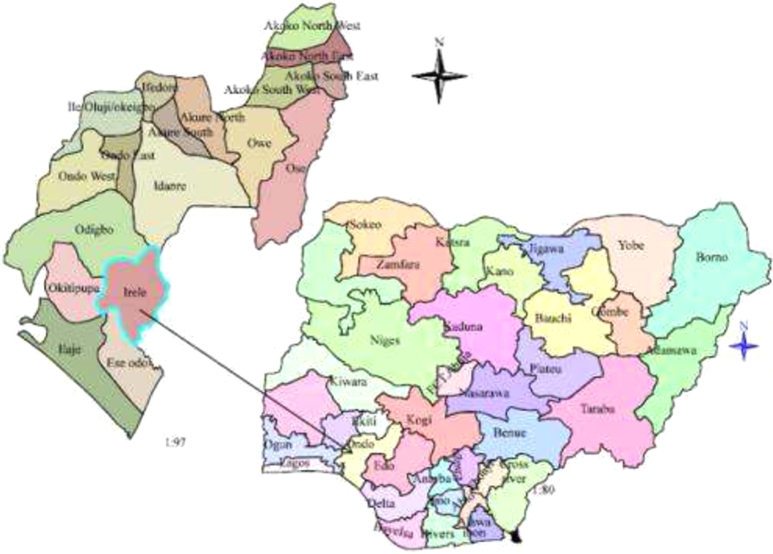
Map of Ondo State.

The study was conducted in Ondo State located in the southwestern part of Nigeria (Fig. 1). The State covers an area of 14,788,723 square kilometres, between latitudes 50 45̀ and 70 52̀N and longitudes 40 20̀ and 60 05̀E. The State is bordered by Ekiti and Kogi States to the north; east to Edo, and to the West by the States of Ogun and Osun. There are three distinct ecological zones within the State. These are the mangrove forest in the south, the rainforest in the central belt, and the savannah in the north. The State has annual rainfall ranging from 1,200 mm in the north to 2,000 mm in the south with the rainy season between March and October and the dry season (November - March) with a temperature of 21-29 degrees throughout the year. Annual rainfall varies from 2000 mm in the southern areas to 1150 mm in the Northern areas. The population of the State of Ondo is estimated at 4,671,700 (NPC, 2022). Cocoa cultivation is one of the main crops grown in the State and is dominated by smallholder cocoa farmers (Omosuyi et al., 2021). The study focused on only cocoa farmers and the data collected were for the 2021/2022 cropping season. The data were collected between October and November 2021 through personal interviews.

### 3.2 Sampling procedure and sample size

A multistage sampling procedure was used to select respondents for the study. The first stage involved the purposive selection of six (6) Local Government Areas (LGAs) due to the prevalence of cocoa production in LGAs. The LGAs are Idanre, Ose, Akure north, Ile-oluji/Okeigbo, Owo, and Akure south. The second stage involved the purposive selection of four (4) communities from each selected local government area based on the predominance of cocoa farmers in the communities. In the third stage, ten (10) smallholder cocoa farmers in each community were selected using a simple random technique. A total of 240 smallholder cocoa farmers were used for the study.

### 3.3 Analytical techniques and model

Data were analyzed with the aid of descriptive statistics, a double hurdle regression model, and an Endogenous switching probit regression model.

### 3.4 Double hurdle model

#### 3.4.1 Theoretical double hurdle model

To analyze the determinants of access to trade credit and the amount of trade credit obtained, a two-decision process model is suitable to use. Most times, the Tobit model is frequently used under the assumption that the two decisions are jointly affected by the same set of factors. However, the Tobit model relies on a single mechanism that is, corner solution application, to determine the two-decision process. This limits the use of the Tobit model in evaluating two-decision processes. Given this limitation, a Double-Hurdle model has been implemented by several studies (Tefera et al., 2014; Beshir, 2014; Hazarika et al., 2016; Anang and Yeboah, 2019; Adeyemo and Kehinde, 2019; and Adeyemo and Kehinde, 2020), was adopted for the study. The Double-Hurdle model assumes that the decision to access trade credit precedes the decision on the amount of trade credit obtained by the smallholder cocoa farmers and that the factors affecting the two decisions are likely to be different. This could be attributed to the fact that the socio-economic features of the farmers are heterogeneous across individuals (Hazarika et al., 2016). In this study, the double-hurdle model was used to determine factors influencing the decision to access trade credit and the amount of trade credit obtained by the smallholder cocoa farmers. This two-decision process is conditioned on some socio-economic features of the farmers.

The first hurdle of the double-hurdle model has access to the trade credit (D) decision with the following equation (1):

(1) $$\begin{array}{c} D_{i}=1....\text{if}...D_{i}^{⁎}>0...\;\text{and}\\ D_{i}=0....\text{if}...D_{i}^{⁎}\leq 0\\ D_{i}^{⁎}=\alpha^{\prime }Z_{i}+\varepsilon_{i} \end{array}$$

where $D_{i}^{⁎}$ is a latent variable that takes the value 1, if the farmer access trade credit or 0, if otherwise, $Z_{i}$ is a vector of household characteristics and *α* is vector of parameters. $\varepsilon_{i}$ refers to the standard error term.

The second hurdle expresses the amount of trade credit obtained by the respondents. The second hurdle equation was estimated using a regression truncated at zero (similar to a Tobit model) which considers all the non-zero (positive) observations of the first hurdle (equation (2)).

The truncated model is expressed as:

(2) $$\begin{array}{c} Y_{i}=Y_{i}^{⁎}\;\text{if}\;Y_{i}^{⁎}>0\;\text{and}\;D_{i}^{⁎}>0\\ Y_{i}=0\;\text{if}\;\text{otherwise}\\ Y_{i}^{⁎}=\beta^{\prime }X_{i}+V_{i} \end{array}$$

where, $Y_{i}^{⁎}$ is the amount of trade credit obtained by the respondents, $X_{i}$ is a vector of explanatory factors, *β* is a vector of parameter and $V_{i}$ is the standard error term.

The error terms are distributed as shown in equation (3):

(3) $$\left\{ \begin{array}{c} \varepsilon_{i}∼N(0,1)\\ V_{i}∼N(0,\sigma 2) \end{array}\right.$$

The error terms $\varepsilon_{i}$ and $V_{i}$ are usually assumed to be independently and normally distributed. It is assumed that for each respondent, the decision to access trade credit and the decision on the amount of trade credit obtained by the smallholder cocoa farmers are made independently.

Finally, the observed variable in a Double-Hurdle model is shown in equation (4):

(4) $$Y_{i}=D_{i}^{⁎}Y_{i}^{⁎}$$

The log-likelihood function for the Double-Hurdle model is shown in equation (5):

(5) $$\text{LogL}=\sum_{0}\ln\left[1-\phi \alpha z_{i}\left(\frac{\beta X_{i}^{\prime }}{\sigma }\right)\right]+\sum_{+}\ln\left[\phi \alpha z_{i}\frac{1}{\sigma }\phi \left(\frac{y_{i}-\beta X_{i}^{\prime }}{\sigma }\right)\right]$$

#### 3.4.2 Empirical double hurdle model

The first hurdle (Probit model) for smallholder cocoa farmers' decision to access trade credit estimated in the empirical double hurdle model is specified as follows in equation (6):

(6) $$P_{i}=\gamma_{0}+\gamma_{1}\text{AGE}+\gamma_{2}\text{HHSIZE}+\gamma_{3}\text{YYEDU}+\gamma_{4}\text{YYEXP}+\gamma_{5}\text{FARSIZE}\;\;+\gamma_{6}\text{GEND}+\gamma_{7}\text{EXTVISIT}+\gamma_{8}\text{ACCREDIT}+\gamma_{9}\text{COOP}+\upsilon_{i}$$

where $P_{i}$ measures the *i*^th^ smallholder cocoa farmers' decision to access trade credit,

The explanatory variables are: AGE = Age of respondent (years); HHSIZE = Household size (actual number); YYEDU = Years spent in school; YYEXP = Years of farming experience; FARSIZE = Farm size (hectare); GEND = Gender (1 = male; 0 = female); EXTVISIT = Extension contacts (1 = yes; 0 = no); ACCASST = Asset (); COOP = membership of cooperatives (1 = member; 0 = otherwise); *υ* = error term.

Empirically, the truncated regression model is specified for this study as follows in equation (7):

(7) $$Y=\beta_{0}+\beta_{1}\text{AGE}+\beta_{2}\text{HHSIZ}+\beta_{3}\text{YYEDU}+\beta_{4}\text{YYEXP}+\beta_{5}\text{FAM}\;\;+\beta_{6}\text{GEND}+\beta_{7}\text{EXTENVIS}+\beta_{8}\text{ACCDRE}+\beta_{9}\text{COOPMEM}+\mu$$

where *Y* is the amount of trade credit obtained by the *i*^th^ smallholder cocoa farmer,

The explanatory variables are: AGE = Age of respondent (years); HHSIZE = Household size (actual number); YYEDU = Years spent in school; YYEXP = Years of farming experience; FARSIZE = size of farm (hectare); GEND = Gender (1 = male; 0 = female); EXTVISIT = Extension contacts (1 = yes; 0 = no); ACCASST = Asset (); COOP = membership of cooperatives (1 = member; 0 = otherwise); *μ* = error term. This study incorporates the independent variable based on the review of existing literature.

### 3.5 Endogenous switching probit model (ESPM)

The decision to use trade credit is modelled in a random utility framework. Let $T_{i}^{⁎}$ denote the difference between the utility derived from using trade credit ($T_{1i}^{⁎}$) and that one derived from not using trade credit ($T_{0i}^{⁎}$), such that a household i will choose to use trade credit, if $T_{i}^{⁎}=T_{1i}^{⁎}-T_{0i}^{⁎}>0$. However, this difference is unobservable, but can be expressed by a latent variable model as follows in equation (8):

(8) $$T_{i}^{⁎}=\gamma X_{i}+\varepsilon_{i}>0\text{with}T_{i}=1\text{if}T_{i}^{⁎}>0$$

where $T_{i}=1$ if a farmer uses trade credit and $T_{i}=0$ otherwise; $X_{i}$ refers to a vector of variables (e.g. gender, age, household size, years of education, farm size among others) that may affect the use of trade credit; *γ* is a vector of parameters to be estimated; and $\varepsilon_{i}$ is an error term, which is assumed to be normally distributed with zero means. Farmers could make use of trade credit to purchase improved inputs such as fertilizers, pesticides, and seeds among others to enhance their productivity, resulting in different adoption rates of improved technologies between users and non-users of trade credit.

For analysis, let $Y_{i}^{⁎}$ represent the net benefits acquired from adopting EU-approved pesticides, the study observes $Y_{i}$, if the underlying latent variable $Y_{i}^{⁎}$ exceeds a certain threshold. Given our interest in exploring the impact of access to trade credit on EU-approved pesticides adoption, while controlling for other factors that may influence farmers' decisions to adopt, I express the decision to adopt EU-approved pesticides as a latent variable function in equation (9):

(9) $$Y_{i}^{⁎}=\beta Z_{i}+\eta T_{i}+\mu_{i}\;\text{with}\;Y_{i}=1\;\text{if}\;Y_{i}^{⁎}>0$$

where $Y_{i}^{⁎}$ is a latent variable that represents the propensity to adopt EU-approved pesticides for household, i, which gives the value of 1, if the farmer adopts EU-approved pesticides and 0 otherwise; $Z_{i}$ is a vector of observable characteristics (e.g. age, education, household size among others) that are assumed to influence EU approved pesticides adoption; $T_{i}$ is an indicator representing the farmer's binary choice of trade credit usage, and *η* are parameters to be estimated and $\mu_{i}$ is a random error term. Considering that farmers themselves decide (self-selection) whether to use trade credit, the coefficient *η* captures the impact of access to trade credit on EU-approved pesticide adoption. The PSM method could be used to account for such selection bias. However, PSM addresses selection bias depending on observable factors. When there are unobservable factors such as farmers' innate abilities that simultaneously influence farmers' decisions to use trade credit and their EU-approved pesticides adoption, the PSM approach may still result in biased estimates. In light of the above, the study employs an ESP model to address sample selection issues (Lokshin and Sajaia, 2011; Ma and Abdulai, 2019; Bello et al., 2021).

ESP model consists of two stages. The first stage addresses farmers' decisions to adopt trade credit using a probit model. In the second stage, a probit model is used to investigate the relationship between EU pesticide adoption and a set of explanatory variables conditional on the use of trade credit. Following Ma and Abdulai (2019), the two outcome equations, conditional on the use of trade credit, can be expressed as follows in equation (10):

(10) $$\begin{array}{c} Y_{1i}^{⁎}=\alpha_{1}X_{1i}+\nu_{1i}\;\text{with}\;Y_{1i}=\left\{ \begin{array}{c} 1\;\text{if}\;Y_{1i}^{⁎}>0\\ 0\;\text{if}\;Y_{1i}^{⁎}\leq 0 \end{array}\right.\text{if}\;T_{i}=1\\ Y_{0i}^{⁎}=\alpha_{0}X_{0i}+\nu_{0i}\;\text{with}\;Y_{0i}=\left\{ \begin{array}{c} 1\;\text{if}\;Y_{1i}^{⁎}>0\\ 0\;\text{if}\;Y_{1i}^{⁎}\leq 0 \end{array}\right.\text{if}\;T_{i}=0 \end{array}$$

where $Y_{1i}^{⁎}$ and $Y_{0i}^{⁎}$ are two latent EU pesticides adoption variables for users and non-users of trade credit, respectively; $Y_{1i}$ and $Y_{0i}$ are observed adoption choices, which take the value of 1 if users and non-users of trade credit adopt the EU pesticides, and 0 if otherwise; Xi is a vector of observable variables (e.g. age, education, household size among others) that affect the decision to adopt EU pesticides; $\alpha_{1}$ and $\alpha_{0}$ are parameters to be estimated; $\nu_{1i}$ and $\nu_{0i}$ are two error terms that represent unobservable factors related to EU pesticides adoption for users and non-users, respectively. The full information maximum likelihood approach estimates the selection Equation (9) and outcome Equations (10) simultaneously (Ayuya et al., 2015; Lokshin and Sajaia, 2011).

For ESP model identification, a variable representing whether a farmer has access to information about trade credit is used as an instrumental variable. To test the validity of the access to trade credit variable as an instrument, we run simple probit models for the use of trade credit and the EU pesticide adoption with the inclusion of the instrumental variable as a regressor. The results, which are presented in the result and discussion section, show that the coefficient of the social organization membership variable is positive and significant in the use of trade credit, but statistically insignificant in EU pesticide adoption. Furthermore, Pearson correlation analysis and sargan test of overidentification also reveals that access to trade credit information is significantly correlated with the use of trade credit, but uncorrelated with EU pesticide adoption. The findings confirm the validity of the access to trade credit information variable as an instrument.

In addition to exploring important factors that influence farmers' decisions to use trade credit and the determinants of EU pesticide adoption separately for users and non-users of trade credit, we are interested in the treatment effects of the use of trade credit on EU pesticide adoption. In particular, the average treatment effects on the treated (ATT) and average treatment effects on the untreated (ATU) are of interest. Following Lokshin and Sajaia (2011), the ATT and ATU can be calculated as follows in equation (11):

(11) $$\begin{array}{c} \text{ATT}=\frac{1}{N_{1}}\sum_{i=1}^{N_{1}}\mathrm{Pr}(Y_{1}=1|T=1,X=x)-\mathrm{Pr}(Y_{0}=1|T=1,X=x)\\ \text{ATU}=\frac{1}{N_{0}}\sum_{i=1}^{N_{0}}\mathrm{Pr}(Y_{1}=1|T=0,X=x)-\mathrm{Pr}(Y_{0}=1|T=0,X=x) \end{array}$$

where $N_{1}$ and $N_{0}$ represent the sample numbers of users and non-users of trade credit, respectively; $\mathrm{Pr}(Y_{1}=1|T=1,X=x)$ and $\mathrm{Pr}(Y_{0}=1|T=0,X=x)$ are predicted probabilities of EU pesticides adoption for users and non-users of trade credit in an observed context, while $\mathrm{Pr}(Y_{0}=1|T=1,X=x)\text{and}\mathrm{Pr}(Y_{1}=1|T=0,X=x)$ are predicted EU pesticides probabilities for those two groups of farmers in a counterfactual context, respectively.

This study incorporates an independent variable based on a review of existing literature (Table 1).

**Table 1:** Description of variables.

Variables	Unit	Expected sign	Description
Gender	Dummy	+	1 = male
			0 = female
Age	Year	+	Measured in years
Household size	Number of persons	+	Measured in number of household members
Education	Years spent in school	+	Measured in years spent in school
Farming experience	Years spent in farming	+	Measured in years spent in farming
Land Ownership	Dummy	+	1 = if farmer owns land
			0 = otherwise
Farm size	Hectares	+	Measured in hectares
Extension service	Dummy	+	1 = if farmer has access to extension service
			0 = otherwise
Cooperative membership	Dummy	+	1 = if farmer belongs to cooperative
			0 = otherwise
Asset	Dummy	+	1 = if farmer owns asset
			0 = otherwise

### 3.6 Specification checks

Access to trade credit and proposed trade credit instruments were compared using correlation analysis. The instrument employed as the instrumental variable for access to trade credit in the ESP model had the highest correlation coefficient with access to trade credit and was uncorrelated with the usage of EU-approved pesticides. The proposed instruments include trade credit information, cooperative membership, access to extension services as well as social organization membership. The rationale behind the selection of these variables was based on the review of the literature. According to Ninh and Kieu (2019), access to information on trade credit, cooperative membership, access to extension services and social organisation membership influence farmers' access to trade credit and might not significantly influence the dependent variable in question (use of EU-approved pesticide). Furthermore, following Cawley and Meyerhoefer (2012), Howley et al. (2015) and Kehinde and Ogundeji (2022b), the Sargan over-identification test was also conducted for the IV models. If the P-value is not significant, it means that the instrument is not correlated with the error term and therefore it is valid (Kehinde et al., 2021).

## 4 Results and discussion

### 4.1 Socio-economic characteristics of smallholder cocoa farmers

Table 2 presents information about the socioeconomic characteristics of respondents. The majority of farmers (85%) are men, suggesting that men might dominate the production of cocoa. This finding is in line with the cultural belief that cocoa farming is a male oriented and dominated enterprise in Southwestern Nigeria. Women only assist in processing of cocoa. This is supported by the report of Ajani and Ashagidigbi (2008), that patriarchal marriages where the base of family power rests with males are common in Ondo State. This might be perhaps due to their physical strength and industrious ability (Oluwatayo et al., 2008; Ayantoye, 2009). The males also have easy access to land especially, where majority of them are the heads of their respective households, the African culture recognizes male child as the only one entitled to inheritance. This is in consonance with findings of Agom et al. (2012) and Oluyole and Sanusi (2009). The smallholder farmers in the study area are 44 years old on the average. This may suggest that the typical farmer in the study area is a young person. This may be explained by the fact that younger individuals are returning to their ancestral villages to invest in farming as a result of a shortage of white-collar jobs. This finding contradicts the expression of Oriola (2009); Agom et al. (2012); Agulanna et al. (2013); Otunaiya and Ibidunni (2014); Oluwatayo (2015) and Kehinde et al., 2018 that the average cocoa farmer in Southwest is old. About 80% of the sampled farmers are married. This finding corroborates the fact that cocoa cultivation is a family enterprise in Nigeria. This confirms earlier findings by various researchers such as Adeyemo et al. (2020) that cocoa production is primarily practiced by married farmers in Southwestern Nigeria. The average household size is 6 people. This implies that most of the households are medium-sized households. This could imply that households have enough helping hands during peak farming activities, which incidentally coincided with the vacation period of school children, to assist in the processing (such as breaking of pod, fermentation and drying) of the cocoa beans. This is explained by the fact that multiple family members can live together and participate in the household's economic activities thanks to the communal aspect of African culture. The outcome is consistent with Alao et al. (2020).

**Table 2:** Socio-economic characteristics of smallholder cocoa farmers.

Variables	Cocoa Farmers
Male (%)	85
Age (years)	44.10(±11.50)
Married (%)	66.7
Household size (#)	6.40 (±3.20)
Formal education (%)	93.4
Years of farming experience	15.90(±8.24)
Farm size (ha)	2.70(±1.09)
Extension Agent (%)	69.2
Access to trade credit (%)	79.2

Source: Field survey, 2020.

Figures in parentheses represent the standard deviation.

About 93.4 percent of respondents have a formal education. This suggests that farmers who grow cocoa are educated individuals. This is a good pointer to their ability to understand and adopt innovation that can improve their efficiency and productivity. This might be explained by the fact that education is a tool that makes individuals effective at whatever activity they are undertaking. The result is in line with Oluyole and Sanusi (2009), and Kehinde and Tijani (2021a) that literate farmers participate in cocoa production in Southwestern Nigeria. Farmers have an average of 16 years of farming experience. This suggests that the farmers have extensive knowledge of the cocoa-growing process. This demonstrates that the farming households have been involved in the cocoa business for a considerable amount of time, which could help them to develop a better understanding of the crop and a mastery of effective farming techniques that might increase their output (Kehinde and Adeyemo, 2017; Adeyemo et al., 2020; Kehinde and Tijani, 2021a). The average farm size of the respondents is 2.70 hectares. This shows that the farmers are smallholders. The finding reaffirms that cocoa production takes place on smallholdings and a large number of cocoa farmers in the Southwest are smallholders. This finding relates to the study of Adeyemo et al. (2020) and Kehinde and Tijani (2021a) that cocoa production in Southwestern Nigeria takes place on smallholdings. About 69.2% of cocoa farmers receive visits from extension agents. This implies information about new technologies in cocoa production will be properly disseminated among the farmers. This could be ascribed to the fact that extension services keep farmers abreast of new farm technologies (Alao et al., 2020). Most of the respondents (79.2%) have access to trade credit. This implies that a large number of cocoa producers have access to trade credit. This result suggests that trade credit could avail the farmers' opportunity to secure EU-approved pesticides to improve the quality of their cocoa beans.

### 4.2 Sources of trade credit

**Figure 2:**
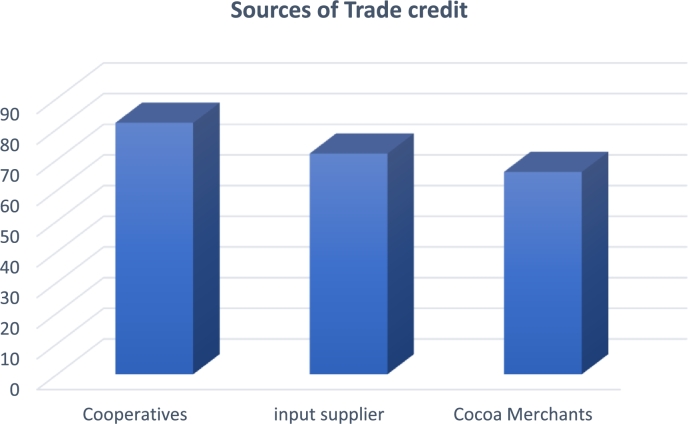
Sources of trade credit. *Multiple responses.

The various sources of trade credit available to smallholder cocoa farmers are presented in Fig. 2. Most respondents obtain trade credit from their respective farmers' cooperative societies (82%), while others obtain trade credit from input suppliers (73%) and cocoa exporters (66%). This implies that there are at least three sources of trade credit available to cocoa farmers, whereas most smallholder cocoa farmers obtain trade credit from their respective cooperatives. According to the finding, cooperatives improve smallholder cocoa farmers' access to trade financing and lower input suppliers' risk. This may be explained by the fact that cooperatives lower the danger of moral hazard and guarantee farmers better prices when purchasing goods (Ninh and Kieu, 2019).

### 4.3 EU approved pesticides usage by cocoa farmers

**Figure 3:**
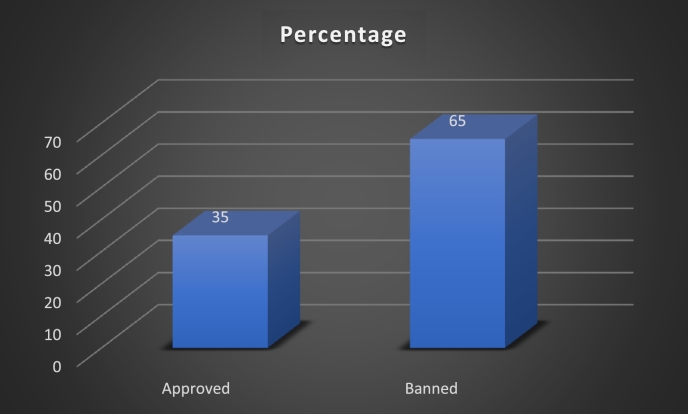
Probability of pesticide usage. Source: Field survey, 2020.

In this study, EU-approved pesticide users are defined as farmers who have applied EU-approved pesticides to their cocoa crops. Only 84 (35%) smallholder cocoa farmers have applied EU-approved pesticides to their cocoa crops (Fig. 3). This implies that the use of EU-approved pesticides is low in the State and farmers still use banned pesticides. This could be attributed to the fact that the banned pesticides are cheap and readily accessible to farmers. This result is in line with the findings of Tijani (2006), Mokwunye et al. (2012), Mokwunye et al. (2014), and Kehinde and Tijani (2021a).

The study further describes the type of EU-approved pesticides used by smallholder cocoa farmers (Table 3). It is noted that the majority (73.4%) of the respondents used the Ridomin 66WP gold (Cuprous Oxide + metalaxyl-M). Others use Esiom 150SL (Acetamiprid) (37.5%), Funguran-OH (Copper hydroxide) (49.1%), touch down fort (Glyphosate) (47.5%), Actara 25WG (Thiamethoxan) (25%). This implies that most of the smallholder cocoa farmers prefer 66WP Ridomin gold (Cuprous Oxide + metalaxyl-M) for a reason. This could be attributed to the fact that there is a high infestation of a fungus, *Phytopthora palmivora*, in the study area, that causes black pod and Ridomin gold (Cuprous Oxide + metalaxyl-M) is effective in mitigating the infestation of the fungus on pods.

**Table 3:** Profile of EU-approved pesticides used.

Trade name	Active Ingredients	Percentage
Esiom 150SL	Acetamiprid	37.5
Funguran-OH	Copper hydroxide	49.1
Touch down fort	Glyphosate	47.5
Actara 25 WG	Thiamethoxan	25.0
Ridomin gold 66WP	Cuprous Oxide + metalaxyl-M	73.4

Source: Field survey, 2020.

*Multiple responses.

### 4.4 Determinants of access and amount of trade credit

The double hurdle regression results of determinants of access to trade credit and the amount of trade credit obtained by cocoa farmers are presented in Table 4. Tests of significance of the model (log-likelihood = −2272) support the use of the DH model as they indicate that the model is of best fit. This suggests that decision to access trade credit and the amount of trade credit accessed are directed by two independent processes and therefore should be analyzed separately. The Double-Hurdle model is statistically significant (p = 0.000) with Wald chi^2^ value of 94.87 indicating a good fit of the model. The first hurdle reveals the determinants of access to trade credit by smallholder cocoa farmers using Probit regression model, while the second hurdle shows the determinants of the amount of trade credit obtained by smallholder cocoa farmers using the Truncated regression model. Probit regression results show that among the socio-economic characteristics of the farmers included in the model, age, gender, household size, farm size, cooperative membership and assets significantly influence the smallholder cocoa farmers' access to trade credit. The coefficients of gender, household size, farm size, cooperative membership, and asset are positive and significant to the smallholder cocoa farmers' access to trade credit. On the contrary, the coefficient of age has a negative significant influence on the smallholder cocoa farmers' access to trade credit. Further to this, it is clear from the results that an increase in household size, farm size, and membership in a cooperative society as well as being male and young farmers would enhance smallholder cocoa farmers' access to trade credit.

**Table 4:** Determinants of access and amount of trade credit.

Variable	First hurdle (probability of accessing trade credit)	Second hurdle (amount of trade credit)
	Coefficient (Z value)	Coefficient (Z value)
Age	−0.111**(−1.98)	1.356(1.01)
Gender	0.369**(2.56)	6.769**(2.17)
Household size	0.043***(2.98)	5.607**(2.05)
Years of education	−0.264(−1.43)	2.395(0.87)
Years of experience in cocoa farming	0.632(0.64)	7.954***(5.49)
Farm size	0.130**(2.28)	1.763(0.12)
Cooperative membership	0.875***(5.03)	7.090**(2.33)
Access to Extension service	−0.086(−0.38)	5.322(1.41)
Asset value	0.601***(2.98)	7.856***(3.00)
Constant	0.013**(2.03)	1.618**(2.15)
No of Obs	240	240
Wald chi^2^ (18)	94.87	
Pseudo R^2^	0.293	
Prob > chi^2^	0.000	
Log-likelihood	−2272	
Ln sigma Constant	1.173***(6.90)	

***, ** &* *represent significance levels at 1%*, 5%&10%, respectively.

Source: Field survey, 2020.

It is reasonable to argue that older farmers are less productive because they are less able to work efficiently or invest in agricultural-related activities as they get older. Therefore, there is a negative correlation between farmers' age and the likelihood that smallholder cocoa farmers will have access to trade credit (Kuwornu et al., 2012 Chandio et al., 2020). Due to this, they are less likely to engage in economic activity or obtain useful resources like trade credit. Additionally, elderly farmers tend to be less risk-averse and use less trade credit to avoid defaulting. The plausible reason for the positive relationship between gender and the probability of smallholder cocoa farmers having access to trade credit is that there is freedom of male mobility in participating in field days and other extension services as compared to the females, which invariably gives them more access to information on trade credit. The male farmers are also very much more capable of resources such as land than their female counterparts which could serve as collateral security in accessing the trade credit. The likely explanation for the association between household size and the likelihood that smallholder cocoa farmers will have access to trade credit is that more people living in the household will put more strain on the limited resources of the household and increase consumption pressure (Nuryartono, 2007; Oyedele et al., 2009 and Chandio et al., 2020).

In this regard, farmers with large families would be required to utilize some of their loans for domestic purposes in order to relieve the pressure on consumption brought on by a large family. Hence, they may default on bank loan repayment. However, in a bid to satisfy the increased household consumption, a farmer tends to seek alternative access to bank credit, which is trade credit to improve his productivity. The likely explanation for the correlation between farm size and the likelihood that smallholder cocoa farmers will have access to trade credit is that larger farms account for a larger portion of the farmer's income, which encourages the farmers to use trade credit to buy EU-approved pesticides to increase productivity (Sebopetji and Belete, 2009; Khan and Hussain, 2011; Akudugu, 2012; Akpan, 2013; Koirala et al., 2016; Chandio et al., 2020). The likelihood that smallholder cocoa farmers will have access to trade credit is positively correlated with membership in cooperative organizations, which may be explained by members' access to trade credit information. The conclusion suggests that farmers are becoming more conscious of trade credit due to their membership in cooperative societies and the idea-sharing among group members. This is accomplished through a number of group-based training programs run by cooperative societies (Kehinde and Tijani, 2021b). In order to advance trade credit, cooperative groups also offer social collateral to farmers, particularly smallholders (Ninh and Kieu, 2019). The plausible reason for the positive relationship between the value of assets and the probability of smallholder cocoa farmers having access to trade credit is that asset ownership represents the social status of the farmers, especially in an African setting, which could enhance their access to trade credit.

In the second hurdle, socio-economic characteristics such as gender, household size, years of farming experience, cooperative membership and assets are significant in influencing the amount of trade credit obtained by smallholder cocoa farmers (Table 5). However, the coefficients of gender, household size, years of farming experience, cooperative membership and assets all had positive signs. This implies that a male farmer with many years of farming experience and a large household and farm, whose also a member of a cooperative would likely have access to a higher amount of trade credit. The plausible explanation for the relationship between gender and the amount of trade credit could be attributed to the fact that male farmers often take risks more than their female counterparts. This is based on the traditional African belief that men are not content with an average lifestyle and this may push them into using more amount of trade credit to fight for surplus production for societal respect and status. The plausible reason for the positive relationship between household size and the amount of trade credit obtained by smallholder cocoa farmers may be traced to the fact that an increase in consumption pressure from large household members may call for an increase in the amount of trade credit which is an alternative to bank credit or personal savings as discussed earlier. The plausible reason for the positive relationship between cooperative membership and the amount of trade credit obtained by smallholder cocoa farmers is traced to the fact that continual educational training programmes in cooperative societies may increase the amount of trade credit assessed by farmers. The likelihood that a smallholder cocoa farmer will earn more trade credit as his social status rises is a feasible explanation for the positive correlation between the asset's worth and the quantity of trade credit they receive. The most logical explanation for the association between smallholder cocoa farmers' trade credit usage and agricultural experience is that a farmer with more experience is likely to have gained knowledge and skills for utilizing newer technologies. The results are consistent with the findings of Amjad and Hasnu (2007), Sebopetji and Belete (2009), Obilor (2013), Duniya and Adinah (2015), Chandio et al. (2020).

**Table 5:** Correlation values of instrumental variables with access to trade credit and Use of EU-approved pesticides.

Variables	Trade credit information	Membership in social organization	Access to extension service	Cooperative membership
Access to trade credit	0.657	0.612	0.366	0.325
	(0.000)	(0.001)	(0.002)	(0.003)
Remarks	Significant	Significant	Significant	Significant
Use of EU-approved pesticides	0.782	0.680	0.313	0.368
	(0.447)	(0.901)	(0.00)	(0.128)
Remarks	Not significant	Not significant	Significant	Not significant

***, ** &* *represent significance levels at 1%*, 5%&10%, respectively.

Source: Field survey, 2020; Figures in parenthesis are the p-values.

### 4.5 Are instrumental variables valid?

#### 4.5.1 Correlation test

An examination of the connection between having access to trade credit and using EU- approved pesticides was done using the suggested instruments to determine the validity of the instrumental variables utilized in the ESP model. The suggested instruments included membership in cooperatives, access to extension services, access to trade credit information, and membership in social organizations. The results of the correlation analysis are presented in Table 5. Access to trade credit information has significant correlations with access to trade credit, but an insignificant correlation with the use of EU-approved pesticides. It also has the highest significant correlation coefficient (0.657) with access to trade credit.

#### 4.5.2 Regression test

The IVs must meet two requirements in order to be considered valid instruments: 1) they must have an impact on the factors that determine access to trade credit, which is a nontrivial function of instruments; and 2) they must not directly affect the use of EU-approved pesticides, but rather only have the potential to do so through the possibility of access to trade credit conditional on covariates. Four instrumental variables—access to trade credit information, membership in cooperatives, use of extension services, and participation in social organizations—were used to instrument the availability to trade credit. All four variables are generally assumed to be exogenous to access to trade credit. Furthermore, because the validity of the IVs is a major challenge in the identification strategy of our model as it is not a testable hypothesis, I ran a probit regression model where the dependent variable takes a value of 1 if a cocoa farmer has access to trade credit; otherwise, zero. Table 6 provides the results of the validity tests of the instruments. The result further establishes the strength of access to trade credit information as a determinant of access to trade credit. All four instruments except access to extension service were jointly significant at the 1 percent level. The chi-square statistics value in the first stage regression is 308.27 (p value = 0.000), which satisfies the theoretical relevancy requirement for instrument validity. Table 6 also reports the *p*-value results for testing the null hypothesis that IVs affect the use of EU-approved pesticides. Another issue with IVs is their strength. I conducted empirical research into the weak instrumental variables issue and found that our IVs are not weak (Kehinde et al., 2021). In addition, I contend that after adjusting for variables, the chosen IV satisfies the exclusion restriction requirements. Additionally, I verified the accuracy of these instruments by carrying out a straightforward fabrication test under the assumption that accurate instruments will affect access to trade credit but not the use of EU- approved pesticides. Access to information trade credit statistically significant determinant of access to trade credit (Chi-square statistics = 299.72 and p-value =<0.001) but not of the use of EU-approved pesticides (F-statistics = 275.13 and p value = 0.000). Access to information on trade credit also has the highest significant coefficient (0.599) with the access to trade credit.

**Table 6:** Regression values of instrumental variables with access to trade credit and Use of EU-approved pesticides.

Variable	Access to trade credit	Use of EU-approved pesticide
Access to credit information	0.599***(5.54)	0.739(0.11)
Cooperative membership	0.340***(3.69)	0.568(0.47)
Access to extension services	0.309(0.81)	0.711**(2.13)
Social organisation membership	0.371***(4.37)	0.882(0.30)
Constant	1.772***(4.40)	1.856***(5.62)
Observations	240	240
Pseudo R-square	0.408	0.449
LR chi2(4)	308.27	275.13
Prob > chi^2^	0.000	0.000

***, ** &* *represent significance levels at 1%*, 5%&10%, respectively.

Source: Field survey, 2020; Figures in parenthesis are the t-values.

### 4.6 Sargan test of instrumental variables

For instrument validation, Sargan's standard over-identification test was also run. If appropriate instruments can be found, the instrumental variable approach is the most effective strategy to take into account all types of endogeneity. However, the proposed instrument must not only be uncorrelated with the dependent variable and error term (valid), but also with the endogenous explanatory variable (Murray, 2006; Burgess et al., 2016). Access to trade credit information was identified using correlation and regression approaches. The next challenge is meeting the necessary requirements of the Sargan test of over-identification. The Sargan standard over-identification test for instrument validation was conducted in this regard. The satisfying condition is that the instrument's p-value must exceed significance values of 0.1, to be a valid instrument (Cawley and Meyerhoefer, 2012; Howley et al., 2015; Cawley et al., 2018; Kehinde and Ogundeji, 2022b). The over-identification test result for Sargan is shown in Table 7, and access to trade credit information is reported to be a legitimate instrument because its p-value is more than the significance threshold of 0.1. As a result, our estimates would be objective and consistent, since we have an instrument i.e access to information on trade credit that is sufficiently accurate to resolve any endogeneity issues that could come from both the use of EU-approved pesticides and access to credit.

**Table 7:** Sargan test of instrumental variable.

Variable	Use of EU-approved pesticide	
	Probit model	2SLS Probit model
Access to trade credit	0.599***(5.54)	0.739***(0.11)
Sargan Estimates		0.848(0.84)

***, ** &* *represent significance levels at 1%*, 5%&10%, respectively.

Source: Field survey, 2020; Figures in parenthesis are the t-values.

### 4.7 Effect of trade credit on EU approved pesticides use among smallholder cocoa farmers

The results from Table 8 show that the full information maximum likelihood estimates of the ESPM are fit for controlling unobserved selection bias in the study. The estimated correlation coefficients for users of trade credit *r*1 and that of non-users of trade credit *r*0 were positive. The positive sign of the covariance term (*r*1) for the correlation between the use of trade credit and the use of EU-approved pesticides suggests a positive selection bias. This shows that the users of trade credit achieve above-average use of EU-approved pesticides regardless of whether they use trade credit or not, but they are better off when they use trade credit. In contrast, non-users of trade credit have a below-average use of EU-approved pesticides in either case but would be better off when they use trade credit. In short, farmers that use trade credit, are more likely to adopt EU-approved pesticides due to unobserved factors. The model is estimated in two parts, first part is the probit model of the determinants of trade credit use. The model reveals that gender, age, household size, farm size, and cooperative membership significantly influence smallholder cocoa farmers' access to trade credit. The coefficients of gender, household size, farm size, and cooperative membership had positive signs. This implies that an increase in any of these variables may increase the smallholder cocoa farmers' access to trade credit. The coefficient of the age had negative signs. This implies that an increase in this variable may decrease the smallholder cocoa farmers' access to trade credit. The plausible explanation for the relationship between these significant variables and access to trade credit has been thoroughly explained in the previous section.

**Table 8:** Impact of the use of trade credit on the adoption of EU-approved pesticides.

Variables	Use of Trade Credit	EU approved pesticides users	
		Users of trade credit	Non-users of trade credit
Gender	0.479***(3.48)	0.172***(3.18)	0.157*(1.86)
Age	−0.426**(−1.99)	−0.418**(−2.17)	−0.280**(−2.41)
Household size	0.223***(3.01)	0.289**(2.19)	0.169(0.35)
Years of education	0.473(1.00)	0.398***(4.74)	0.665***(6.04)
Years of farming experience	0.178(0.36)	0.393**(2.44)	0.277***(4.19)
Land ownership	0.498(0.29)	0.230(0.84)	0.325***(3.92)
Farm size	0.301***(9.94)	0.234(0.29)	0.482***(2.65)
Extension services	0.315(0.59)	0.529***(2.96)	0.632(0.46)
Cooperative membership	0.532**(2.48)	0.637***(3.77)	0.445**(6.67)
Asset	0.381(0.78)	0.239***(3.87)	0.686(0.48)
Constant	0.278***(3.76)	0.205***(3.20)	0.263****(5.25)
P_0_	8.783***(5.29)		
P_1_	9.654***(6.67)		
Wald Chi-square	96.09		
Loglikelihood	−2697.84		
LR test of Indep. Eqns: Chi^2^(2) = 27.54 Prob > chi=20.000			

***, ** &* *represent significance levels at 1%*, 5%&10%, respectively.

Source: Field survey, 2020; Figures in parenthesis are the t-values.

The second stage is the switching probit regression model for use of EU-approved pesticides. The results of the switching probit regressions for use of EU-approved pesticides among users and non-users of trade credit are presented in the third and fourth columns, respectively. The model revealed that age, household size, education, access to extension services, and cooperative membership significantly affect the use of EU-approved pesticides among users of trade credit. In furtherance, the coefficient of household size, education, access to extension services, and cooperative membership had positive signs. This implies that an increase in any of these variables may increase the likelihood of using EU-approved pesticides among users of trade credit. However, the coefficient of age had a negative sign. This implies that an increase in this variable may decrease the likelihood of using EU-approved pesticides among users of trade credit. Meanwhile, among non-users of trade credit, variables such as age, farming experience, farm size, land ownership, and cooperative membership significantly affect the use of EU-approved pesticides. The coefficients of farming experience, farm size, land ownership, and cooperative membership had positive signs. This implies that an increase in any of these variables may increase the likelihood of using EU-approved pesticides among non-users of trade credit. Whereas age had a negative sign. This implies that an increase in this variable may decrease the likelihood of using of EU approved pesticides among non-users of trade credit

The reasonable argument for the negative relationship between the age of the farmers and the use of EU-approved pesticides among smallholder cocoa farmers is that older farmers are more conservative than younger ones to adopt new technology due to their risk-averse nature. Whereas younger farmers are more innovative and consequently may easily try innovative technologies such as EU-approved pesticides. Also, older cocoa farmers are more susceptible to health hazards associated with pesticide spraying compared to younger farmers. Therefore, younger farmers are more likely to adopt new technologies than older farmers (Adejumo et al., 2014). However, there is a positive relationship between education and the use of EU-approved pesticides among smallholder cocoa farmers, which might be attributed to the fact that education gives farmers the ability to comprehend information about new technologies much faster than their counterparts with lower education (Anang and Amikuzuno, 2015). The plausible reason for the positive relationship between household size and the use of EU-approved pesticides among smallholder cocoa farmers is that large families may be forced to use EU-approved pesticides in an attempt to earn more income to ease the consumption pressure imposed by a large household. Household size can also be a proxy for labour supply that could have an impact on driving or constraining adoption. The plausible reason for the positive relationship between farm size and the use of EU-approved pesticides among smallholder cocoa farmers is that large farms encourage farmers to try new technologies on a portion of land without compromising the food security of their household. Similarly, there is a positive relationship between membership in a cooperative society and the use of EU-approved pesticides among smallholder cocoa farmers which might be ascribed to the fact that members in a cooperative society may expose farmers to a wide range of ideas and better access to information, through training and extension services, on EU approved pesticide (Anang and Amikuzuno, 2015). Agricultural groups provide social network platforms within which participants share new information and experiences such as EU-approved pesticide use. The plausible reason for the positive relationship between access to extension services and the use of EU-approved pesticides among smallholder cocoa farmers is that access to agricultural extension services enhances the dissemination of information about the EU-approved pesticides. This could be based on the fact that extension workers transfer knowledge from researchers to farmers and advice farmers on new technologies (Raghu and Manaloor, 2014). The plausible reason for the positive relationship between land ownership and the use of EU-approved pesticides among smallholder cocoa farmers is that land ownership promotes both soil-conserving and yield-enhancing technologies such as EU-approved pesticides. Tenure security associated with land ownership can as well be peculiar to the decision to adopt long-term technologies such as EU-approved pesticides (Oyetunde-Usman et al., 2021). The plausible reason for the positive relationship between farming experience and the use of EU-approved pesticides among smallholder cocoa farmers is that farmers with higher experience are expected to have information and better knowledge to evaluate the advantage of EU-approved pesticides (Kehinde, 2021). The result further reveals that households' social and wealth status in the form of access to assets has an important influence on the use of EU-approved pesticides. This is premised on the fact that households with better access to assets can purchase any improved technology to enhance their productivity. This study agrees with the study of (Kehinde, 2021; Kehinde and Ogundeji, 2022a), which states that the social and wealth status of farmers is one of the important factors that drive the adoption of improved technologies.

In terms of the selection of observables and unobservables, ATU has a coefficient of 0.73 while ATT has a coefficient of 0.89 (Table 9). In other words, average treatment on untreated generates a 73 per cent rise in the adoption of EU-approved pesticides, whereas average treatment on treated causes an 89 per cent increase in the adoption of EU-approved pesticides. Both effects are statistically significant at the 1% level. According to the positive sign of rho, unobservable variables that improve the adoption of EU-approved pesticides correlate with unobservable variables that improve trade credit among the farmers. This means that cocoa farmers are more likely to receive trade credit and invest in the adoption of EU-approved pesticides. Failure to account for it in this scenario will result in an underestimation of the impact of trade credit on the adoption of EU-approved pesticides. As a result, the ESP model produces a larger effect. The ATT is statistically significant, according to the t-test. This means that farmers that receive trade credit adopt EU-approved pesticides more than those who do not. This could be explained by the fact that farmers may tend to afford and purchase more EU-approved pesticides when they have access to trade credit, regardless of the cost of the pesticides or if they are bank credit constrained.

**Table 9:** Results of Impact models.

Variable	Mean	Standard error	T-test
ATT	0.888	0.397	9.05***
ATU	0.730	0.291	6.52***

***, ** &* *represent significance levels at 1%*, 5%&10%, respectively.

Source: Field survey, 2020.

## 5 Conclusion and recommendations

This study investigated the trade credit and its impact on the use of EU-approved pesticides among smallholder cocoa farmers in Ondo State. Data were analyzed using descriptive statistics, double hurdle regression model and an endogenous switching probit regression model. The study concluded that the majority of the smallholder cocoa farmers were male, smallholder, and at their productive age. Most of the respondents (79%) have access to trade credit. Most obtain trade credit from their respective farmers' cooperatives (82%), while others obtain trade credit from input suppliers (73%) and cocoa exporters (66%). It is also noted that only 84 smallholder cocoa farmers use EU-approved pesticides on their cocoa farms. The majority (73.4%) of the respondents used the Ridomin 66WP gold (Cuprous Oxide + metalaxyl-M). Others use Esiom 150SL (Acetamiprid) (37.5%), Funguran-OH (Copper hydroxide) (49.1%), touch down fort (Glyphosate) (47.5%), Actara 25WG (Thiamethoxan) (25%). The result further shows that age, gender, household size, farm size, cooperative membership, and assets significantly influenced the probability of a farmer receiving trade credit. While, gender, household size, year of farming experience, cooperative membership, and assets are statistically significant in determining the amount of trade credit obtained by the farmers. The first stage of the ESPM shows that gender, age, household size, farm size, and cooperative membership significantly influence the smallholder cocoa farmers' access to trade credit. The second stage of the ESPM shows that age, household size, education, access to extension services, and cooperative membership significantly affect the use of EU-approved pesticides among users of trade credit. Meanwhile, among non-users of trade credit, variables such as age, farming experience, farm size, land ownership, and cooperative membership significantly affect the use of EU-approved pesticides. After controlling for observed and unobserved covariates, the study concluded that access to trade credit positively impacts the use of EU- approved pesticides among smallholder cocoa farmers.

Given the above conclusion, the following are recommended:

i. To enhance the uptake of EU-approved pesticides, farmers' education about their use should also be strengthened. Additionally, pertinent organizations like extension agents should step up their efforts to inform farmers about the sources of trade credit facilities that are accessible to them.
ii. The findings also suggest that smallholder cocoa farmers' prospects of receiving trade finance can be increased by joining cooperatives. Therefore, the government should think about implementing an information-driven program to support trade credit through the establishment of strong agricultural institutions like cooperatives that plays a vital role in increasing smallholder cocoa farmers' access to trade credit.
iii. The study also recommends launching a trade credit program through rural innovation platforms to boost the use of EU-approved pesticides in Nigerian cocoa cultivation.
iv. As the EU-approved pesticides are too expensive for farmers to afford, cocoa pesticide traders should step up their trade credit services to cocoa farmers. This will help farmers to utilize more EU- approved pesticides.

## Declarations

### Author contribution statement

Ayodeji Kehinde: Conceived and designed the experiments; Performed the experiments; Analyzed and interpreted the data; Contributed reagents, materials, analysis tools or data; Wrote the paper.

### Funding statement

This research did not receive any specific grant from funding agencies in the public, commercial, or not-for-profit sectors.

### Data availability statement

Data will be made available on request.

### Declaration of interests statement

The authors declare no conflict of interest.

### Additional information

No additional information is available for this paper.
